# Emergence of Hepatitis B Virus Genotype F in Aligarh Region of North India

**DOI:** 10.1155/2013/846849

**Published:** 2013-12-05

**Authors:** Hiba Sami, Meher Rizvi, Mohd Azam, Rathindra M. Mukherjee, Indu Shukla, M. R. Ajmal, Abida Malik

**Affiliations:** ^1^Department of Microbiology, Jawaharlal Nehru Medical College, Aligarh Muslim University, Aligarh 202002, India; ^2^Asian Institute of Gastroenterology, Hyderabad 500082, India; ^3^Department of Medicine, Jawaharlal Nehru Medical College, Aligarh Muslim University, Aligarh 202002, India

## Abstract

*Introduction*. HBV genotypes and subtypes are useful clinical and epidemiological markers. In this study prevalent HBV genotypes were assessed in relation to serological profile and clinical status. *Material & Methods*. 107 cases of HBV were genotyped. Detailed clinical history was elicited from them. HBsAg, HBeAg, anti-HBs, anti-HBe, and anti-HBc-IgM were assessed. HBV genotyping was performed using Kirschberg's type specific primers (TSP-PCR), heminested PCR, and Naito's monoplex PCR. Nucleotide sequencing was performed. *Results*. A total of 97 (91%) were genotyped following the methods of Kirschberg et al./Naito et al. Genotype D was by far the most prevalent genotype 91 (85.04%) in this region. A surprising finding was the detection of genotype F in 5 (4.67%) of our patients. Genotype A strangely was observed only in one case. In 85.7% genotype D was associated with moderate to severe liver disease, 43.9% HBeAg, and 18.7% anti-HBc-IgM positivity. Majority of genotype F (80%) was seen in mild to moderate liver disease. It was strongly associated with HBeAg 60% and 20% anti-HBc-IgM positivity. *Conclusion*. Emergence of genotype F in India merits further study regarding its clinical implications and treatment modalities. Knowledge about HBV genotypes can direct a clinician towards more informed management of HBV patients.

## 1. Introduction

Hepatitis B virus (HBV) is one of the major public health problems worldwide. About 30% of the world population has serological evidence of current and past infection with HBV [1] and approximately 1 million persons die annually from HBV related chronic liver diseases including severe complications such as liver cirrhosis and hepatocellular carcinoma [2]. Every year there are over 4 million acute clinical cases of HBV and about 25% of carriers. Approximately one million people a year die from chronic active hepatitis, cirrhosis, or primary liver cancer [3].

Genotypically, HBV is divided into 8 groups, A–H. HBV genotypes represent naturally occurring strains of HBV that have evolved over the years in the world. The genotypes and subtypes were identified on the basis of intergroup divergence of 8% or 4% in gene (S) sequence, respectively. They are useful clinical and epidemiological markers [4]. It is also well known that genotypes vary geographically and correlate strongly with ethnicity [5]. Genotype correlation has been associated with HBV core antigen, HBe antigen seroconversion, activity of liver disease, and treatment response with chronic HBV infections [6, 7]. Type A is prevalent in Europe, Africa, and southeast Asia, including the Philippines. Types B and C are predominant in Asia; type D is common in the Mediterranean area, the Middle East, and India; type E is localized in sub-Saharan Africa; type F (or H) is restricted to Central and South America. Type G has been found in France and Germany. Genotypes A, D, and F are predominant in Brazil and all genotypes occur in the United States with frequencies dependent on ethnicity. The E and F strains appear to have originated in aboriginal populations of Africa and the New World, respectively.

Currently HBV genotypes can be determined by several methods, including direct sequencing [8], restriction fragment length polymorphism analysis (RFLP) [9], line probe assay [10], PCR using type specific primers [11], calorimetric point mutation assay [12], ligase chain reaction assay [13], and enzyme linked immunosorbent assay for genotype specific epitopes [14]. Kirschberg and Naito primers were used to genotype HBV in Aligarh region of North India. The current study was done to determine the prevalence of various HBV genotypes in Aligarh region in patients of acute and chronic hepatitis by using type specific primers and the clinical and demographic characteristics in relation to their genotypes.

## 2. Material and Methods

### 2.1. Study Group

Three hundred and thirty patients presenting with the sign and symptoms of liver disease were evaluated on the basis of various investigations such as liver function test (AST, ALT, T. Bilirubin, and ALP), S.creatinine, PT/INR, MELD Score, ultrasound, CT scan, and G.I. endoscopy. Detailed clinical history and rigorous physical examination was conducted on them. 107 confirmed cases of HBV, positive for HBsAg, were included in the study. This study was conducted after obtaining permission from institutional ethics committee of J.N. Medical College and the procedure followed in the study was in accordance with institutional guidelines and informed consent was obtained from all the patients before including them in the study. All patients underwent complete physical examination and detailed clinical history was elicited from them.

### 2.2. Exclusion Criteria

Patients with autoimmune hepatitis, alcoholic hepatitis, drug induced hepatitis, patients giving history of recent infection, surgery, trauma within the preceding two months, renal insufficiency, or with other acute or chronic inflammatory diseases were excluded from this study. None of the participants had received any antiviral or immunosuppressive therapy before or during the course of this study.

### 2.3. Serological Investigations

All patients with hepatitis were screened for HAV (hepatitis A virus), HBV (hepatitis B virus), HCV (hepatitis C virus), HEV (hepatitis E virus) and HIV by commercially available ELISA kits: HBsAg, third generation anti-HCV, fourth generation anti-HIV (J. Mitra & Co., Pvt. Ltd., India), anti-HAV IgM, and anti-HEV IgM (DRG International, Inc., USA). HBc lgM antibodies were tested in 107 HBsAg positive samples using DRG Anti- Hepatitis B Core IgM Antigen ELISA kit, (DRG International Inc., USA). The tests were performed according to the manufacturer's instructions. Patients positive for HBV were enrolled for further study. Further on the basis of duration of illness, serum examination, and biochemical examination these patients were regrouped as follows.

*(a)  Acute Hepatitis B*. It is defined as a condition associated with prodromal symptoms preceding the onset of jaundice by 1-2 weeks (e.g., anorexia, nausea, vomiting, and fatigue), fever, an onset of clinical jaundice, diminished constitutional prodromal symptoms, hepatomegaly, jaundice, hyperbilirubinemia (>17 mol/L), serum amino alanine transaminase (ALT) and aspartate amino transferase (AST) at least fivefold greater than normal, HBsAg (+), anti-HBc-IgM (+), anti-HBc IgG (−), and anti-HBs (−).

*(b)  Chronic Hepatitis B*. It is defined as a condition associated with fatigue, anorexia, jaundice, hepatomegaly, density of the liver harder than normal, splenomegaly, hyperbilirubinemia more than twofold higher than in healthy individuals, serum AST, and ALT twofold higher than in the healthy control group, and HBsAg (+) for longer than 6 months, and anti-HBc type IgG (+), anti-HBc type IgM (−).

*(c)  Fulminant Hepatic Failure/Hepatic Encephalopathy*. FHF was diagnosed if the patients developed hepatic encephalopathy within 4 weeks from the onset of acute hepatitis.

All cases of acute HBV infection were followed up for 6 months to assess seroconversion to anti-HBs (DRG International, Inc., USA). All cases which seroconverted to anti-HBS were included in acute HBV (AHB) group and those who were HBsAg (+) for longer than 6 months were included in chronic HBV (CHB) group. 107 confirmed cases were further screened for HBeAg (DRG International Inc., USA) and anti HBc IgM (DRG International, Inc., USA).

#### 2.3.1. Other Investigations

Liver function tests (LFT) like serum amino alanine transaminase (ALT), serum aspartate amino transferase (AST) and alkaline phosphatase (ALP), bilirubin (direct and indirect), total bilirubin, albumin, globulin, creatinine, and international normalized ratio for prothrombin time were performed.

#### 2.3.2. DNA Extraction

Total DNA from 100 *μ*L serum was extracted by standard phenol chloroform isoamyl alcohol method [15].

#### 2.3.3. Genotyping and Sequencing of HBV

All 107 cases were subjected to genotyping. HBV genotyping was performed following three pronged approach; initially all samples were subjected to multiplex PCR [16]. HPSF purified oligonucleotides were obtained from MWG-Biotech, Ebersberg, Germany. The sequences of the HBV genotype specific primers were as follows.

(i) *Genotype A*: HBV-GT1-A-s (nt 2331–2360) 5_-CGG AAA CTA CTG TTG TTA GAC GAC GGG AC-3_; HBV-GT1-A-as (nt 2701–2665) 5_-AAT TCC TTT GTC TAA GGG CAA ATA TTT AGT GTG GG-3_
(ii) *Genotype B*: HBV-GT1-B-s (nt 1470–1491) 5_-CCG CTT GGG GCT CTA CCG CCC G-3_; HBV-GT1-B-as (nt 1660–1633) 5_-CTC TTA TGC AAG ACC TTG GGC AGG TTC C-3_
(iii) *Genotype C*: HBV-GT1-C-s (nt 2706–2741) 5_-CCT GAA CAT GCA GTT AAT CAT TAC TTC AAA ACT AGG-3_; HBV-GT1-C-as (nt 192–165) 5_-AGC AGG GGT CCT AGG AAT CCT GAT GTT G-3_
(iv) *Genotype D*: HBV-GT1-D-s (nt 2843–2870) 5_-ACA GCA TGG GGC AGA ATC TTT CCA CCA G-3_; HBV-GT1-D-as (nt 2990—2966) 5_-CCT ACC TTG TTG GCG TCT GGC CAG G-3_
(v) *Genotype E*: HBV-GT1-E-s (nt 2093–2122) 5_-CTA ATG ACT CTA GCT ACC TGG GTG GGT GTA-3_; HBV-GT1-E-as (nt 2880–2853) 5_-CCA TTC GAG AGG GAC CGT CCA AGA AAG C-3_
(vi) *Genotype F*: HBV-GT1-F-s (nt 2843–2871) 5_-ACA GCA TGG GAG CAC CTC TCT CAA CGA CA-3; HBV-GT1-F-as (nt 109–83) 5_-AGA GGC AAT AGT CGG AGC AGG GTT CTG-3_.

Multiplex PCR was carried out in a total volume of 50_l which contained 25 *μ*L of HotStarTaqTM Master Mix, Qiagen, Hilden, Germany, 1 *μ*L of each sense and antisense primer (10 *μ*mol/L), 8 *μ*L template, and water for a total volume of 50 *μ*L. The thermocycler was programmed to first incubate the samples for 15 min at 95°C, followed by 40 cycles consisting of 94°C for 60 s, 60°C for 60 s, and 72°C for another 2 minutes. After PCR the amplified products were electrophoresed on a 2.5% agarose gel, stained with ethidium bromide, and evaluated under UV light (Biorad, USA). The size of the expected amplified product for each Genotype was: Genotype A: 370 bp, Genotype B: 190 bp, Genotype C: 701 bp, Genotype D: 147 bp, Genotype E: 787 bp, and Genotype F: 481 bp.

Heminested PCR was performed on all the sera which could not be genotyped by the above method. Samples which could not be identified by heminested PCR were subjected to monoplex PCR using the method of Naito et al. [11]. In brief, 10 mL of extracted DNA was subjected to 40 cycles of first round PCR using primers 5′-TCA CCA TAT TCT TGG GAA CAA GA-3′ (nt 2823 2845, universal, sense) and 5′-CGA ACC ACT GAA CAA ATG GC-3′ (nt 685-704, universal, antisense) amplifying a 1063 bp region of S gene [11]. TSP-PCR was performed in two separate mixes A and B utilizing 1 mL of 1st round PCR product and subjecting it to two rounds of PCR cycles (20 cycles each) as described by Naito et al. [11].

*In mix A*, primers specific for genotype A (5′-CTC GCG GAG ATT GAC GAG ATG T-3′ nt 113–134, type A specific, antisense), and genotype B (5′-CAG GTT GGT GAG TGA CTG GAG A-3′ nt 324–345, type B specific, antisense), genotype C (5′-GGT CCT AGG AAT CCT GAT GTT G-3′ nt 165–186, type C specific, antisense) and a common universal sense primer (5′-GGC TCA AGT TCA GGA ACA GT-3′ nt 67–86, types A to C specific, sense) were used.

*In mix B*, a common antisense primer (5′-GGA GGC GGA TCT GCT GGC AA-3′ nt 3078–3097, specific for types D to F, antisense) along with genotype specific primer D (5′-GCC AAC AAG GTAGGA GCT-3′ nt 2979–2996, type D specific, sense), E (5′-CAC CAG AAA TCC AGA TTG GGA CCA-3′ nt 2955–2978, type E specific, sense), and F (52-GTT ACG GTC CAG GGT TCA CA-3 nt 3032–3051, type F specific, sense) was used. Mix A allowed for the specific detection of PCR products for types A (68 bp), B (281 bp), and C (122 bp), and mix B allowed for detection of types D (119 bp), E (167 bp), and F (97 bp).

The amplified product of 10 genotype D samples was purified and sequenced by Macrogen, Inc. (Seoul, Korea), using same primers as were used for PCR. Sequencing reactions were performed in a MJ Research PTC-225 Peltier Thermal Cycler using a ABI PRISM BigDyeTM Terminator Cycle Sequencing Kits with AmpliTaq DNA polymerase (FS enzyme) (Applied Biosystems).

#### 2.3.4. Phylogenetic Analysis

For sequence alignment as well as phylogenetic analysis, we selected the GenBank sequences with the best high scoring matching with HBV reference sequences for each genotype [17]. Sequences, were edited, aligned and analyzed using Clustal W Bioedit software. Genetic distances were calculated using the Kimura two parameter algorithms and phylogenetic trees were constructed by the neighbour joining (NJ) method. To confirm the reliability of the pairwise comparison and phylogenetic tree analysis, bootstrap resampling and reconstruction were carried out 1000 times. Phylogenetic analysis was done using MEGA version MEGA 4 package.

#### 2.3.5. Clinical and Biochemical Grading of Severity of Liver Disease

Disease was classified as mild, moderate, and severe according to the presence or absence of sign and symptoms like icterus, pallor, anorexia, jaundice, nausea, vomiting, splenomegaly, ascites, variceal bleeding, weight loss, and abdominal discomfort. Patients with no sign and symptoms were classified as having mild, those with icterus, pallor, anorexia, jaundice, nausea, vomiting as having moderate, and those with splenomegaly, ascites, variceal bleeding, weight loss, and abdominal discomfort as having severe liver disease. Biochemically the patients in whom all the four parameters (ALT, AST, PT-INR, and MELD) were elevated were classified as having severe, those with elevations in three parameters were classified as having moderate, and those having elevation of only two parameters were classified as having mild liver disease.

### 2.4. Statistical Analysis

Statistical analysis was performed with the IBM SPSS Statistics 19. Results were expressed as means ± standard deviation or as percentages. Means were compared between groups by using the *t*-test, ANOVA (one way analysis of variance) and frequency distributions were compared by using the chi-square test.

## 3. Results

The study group is comprised of 107 HBV infected patients. 52 (48.6%) had acute viral hepatitis (AVH), 32 (30%) had chronic viral hepatitis (CVH), 9 (8.4%) had fulminant hepatic failure (FHF), 13 (12%) were incidentally detected asymptomatic HBsAg positive subjects (IDAHS), and 1 (0.93%) had hepatocellular carcinoma (HCC). The patients (54 men and 36 women) ranged in age from 18 to 70 years. The mean age is 34.58 ± 15.58 years. 48 (44.9%) patients were positive for HBeAg and 21 (19.6%) patients were positive for anti-HBc-IgM antibodies. Of 32 CVH cases, 14 (43.7%) were HBeAg positive, and 18 (56.3%) were HBeAg negative, and 6 (28.6%) were positive for anti-HBc-IgM antibodies.

### 3.1. Genotype Distribution

In our study we found that HBV genotype D was by far the most prevalent genotype 91 (85.04%) in this region. A surprising finding was detection of genotype F in 5 (4.67%) of our patients. Genotype A strangely was observed only in one case. In genotype D patients prevalence of HBeAg was observed in 24 (51%) cases of AVH, 10 (38%) in CVH, 4 (80%) in FHF, and 2 (17%) in IDAHS. Genotype D was found to be associated with moderate to severe liver disease in 78 (85.7%) patients based on clinical grading (Table 1). 17 (18.7%) genotype D patients were positive for anti-HBc-IgM. Of the 18 HBeAg negative cases, HBV DNA was detected in 17 (94.4%) cases pointing to presence of precore mutations.

Using multiplex PCR 53 (49.5%) were genotyped using a modified protocol of Kirschberg et al. [16]. The remaining samples were subjected to heminested PCR [18]. An additional 28 (26%) samples were genotyped by this method. Despite amplification by heminested PCR, 26 samples could not be genotyped. Of these, 20 samples were amplified using monoplex PCR following Naito et al. [11] protocol. 16 (80%) of these samples were genotyped. Thus, a total of 91% were genotyped following methods of Kirschberg et al. [16]/Naito et al. [11]. Genotypes A (1) and F (5) were detected initially by primers of Naito et al. [11] whereas only one of the F genotype was detected by Kirschberg et al. [16] and genotype A was identified only by Naito et al. [11] method.

Genotype F was detected in 1AVH, 1 FHF, and 3 CVH cases and was associated with mild to moderate liver disease in 4 (80%) cases based on clinical and biochemical grading (Table 1). Anti-HBc-IgM positivity was seen in 1 (20%) patient.

The individual with genotype A was associated with high ALT, AST, and PT-INR and was positive for HBeAg and anti-HBc-IgM antibodies (Table 2).

On comparing demographic profile, HBeAg status, and biochemical profile of patients with different HBV genotypes (Table 2), no significant findings were observed in any particular genotype. HBeAg was present in 43.9% in genotype D and 60% in genotype F. Genotype F was associated with mildly elevated levels of ALT while the others were associated with severely deranged levels.

### 3.2. Genotyping of HBV by Direct Sequencing

**Figure 1:**
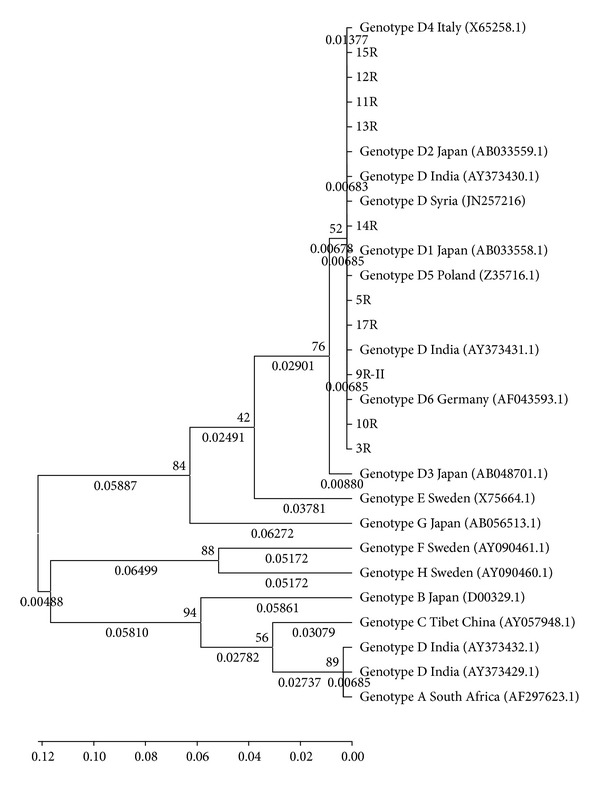
Phylogenetic analysis of isolates 3R, 5R, 9R, 10R, 11R, 12R, 13R, 14R, 15R, and 17R (genotype D) from Aligarh region was compared with reference strains from Genbank; phylogenetic analysis was based on comparison of 305 bp of the S-gene and constructed using neighbour joining method (bootstrap test of phylogeny). Robustness of the phylogenetic analysis was evaluated by a 1000 bootstrap replications. Genetic distances were calculated using the Kimura 2-parameter model of nucleotide substitution, MEGA 4 package.

DNA sequencing and phylogenetic analysis confirmed genotype D in 10 of our representative samples (Figure 1). A clustering of genotype D in our study was observed with genotype D from India and Syria, D4 of Italy, D2 of Japan, D5 of Poland, and D6 of Germany [17].

## 4. Discussion

Different HBV genotypes have their own relatively obvious geographical distribution. HBV genotypes A and D have been well documented from different parts of mainland India [19–22]. In two different studies from northern India, genotypes A and D were found to be equally prevalent [19, 23]. In our study we found that HBV genotype D (85.04%) was by far the most prevalent genotype in the Aligarh region of North India. These results differ from those of two earlier studies in North India where genotypes A and D were found in equal proportions. Contrary to these studies, only one genotype A was identified in our patient group. However, another study from the same region reported genotype D to be predominant (84%) with a low frequency (16%) of genotype A [20]. The pattern of genotype prevalence in this study is in line with studies emanating from Indian subcontinent confirming the high proportion of genotype D in this part of Asia [24]. This study, therefore, highlights the genotypic link between various ethnic groups within the country and people of the neighboring countries.

A surprising finding was detection of genotype F in 4.67% of our patients. Genotype F is considered a new world strain and largely prevalent in central and South America [25, 26]. Genotype F is divided into 4 subgenotypes: F1–F4. Subgenotypes F1 and F2 have been further divided in F1a, F1b, F2a, and F2b [26–29]. In Venezuela subtypes F1, F2, and F3 are found in East and West Amerindians. Among South Amerindians only F3 was found. Subtypes Ia, III, and IV exhibit a restricted geographic distribution (Central America, the North and the South of South America, resp.) while clades Ib and II are found in all the Americas except in the Northern South America and North America, respectively.

One reason for this unexpected finding may be because Aligarh lies in close vicinity to New Delhi which records significant efflux and influx of population to and from different parts of the world. In an era of frequent international travel and human migration, introduction of new HBV genotype to a community might have far reaching effects, including recombination between genotypes or replacement of one genotype by another. However, there is an urgent need to explore other possible reasons for the unusual prevalence of genotype F. In all the five cases the route of transmission appears to be vertical as no other significant factor was identified in any one of them. The patients did not give any history of travel outside India. Prior to this study, Singh et al. [30] have also reported genotype F (3%) from North India. This suggests that genotype F may be indigenous to certain pockets of North India, clustering in and around western UP and Haryana. This unusual finding apparently contradicts the conventional knowledge that HBV genotype closely mirrors ethnic and geographical migration.

In this study Naito primers were more sensitive in detection of HBV genotypes especially genotype F. Naito primers were used when genotyping was unsuccessful using Kirschberg primers. In this way 16 of 20 cases were genotyped. We recommend Naito primers for detection of HBV genotypes. The advantage of Kirschberg of course is that it is multiplex, thus saving time. Heminested PCR using Kirschberg primers further increases sensitivity of these primers.

HBV DNA was detected in 90.6% of our patients using primers of Naito et al. [11] and Kirschberg et al. [16]. HBeAg positivity was seen only in 48 (44.9%) cases. Of 32 CVH cases, 14 (43.7%) were HBeAg positive and 18 (56.3%) were HBeAg negative. Of the 18 HBeAg negative cases, DNA was detected in 17 (94.4%) cases (of which 16 were genotype D and 1 was genotype F), suggesting that due to infection for a prolonged duration these strains may have developed mutations in the precore region. Similar precore mutations were observed in a study in Brazil, in which 58% of the HBV patients were HBeAg negative [31]. Of the 18 HBeAg negative cases, majority had severe liver disease: 3 had hepatic decompensation, 12 had cirrhosis, and 3 were stable. Abdo et al. [32] also reported that HBeAg negativity in genotype D was associated with advancing stages of liver disease as compared to HBeAg positive individuals. Further analysis in large-scale longitudinal studies is required to better delineate this relationship.

Based on clinical grading genotype D was associated with moderate to severe liver disease in 85.7% patients. The prevalence of genotype D in different liver disease groups was 90.1% in AVH, 81.2% in CVH, 55.5% in FHF, 92.3% in IDAHS, and 100% in HCC. Several studies have revealed the association of HBV genotypes with the severity of chronic liver disease, but the results are not consistent [33, 34]. Wai et al. [35] and Rodriguez-Frias et al. [36] reported that genotype D is more prevalent in acute disease, and reported a significant association between genotype D and acute liver failure compared to chronic hepatitis. In contrast, in a study from Egypt, Zekri et al. [37] reported that genotype D was significantly more prevalent in chronic active hepatitis than acute hepatitis. In this study genotype D was associated strongly with all stages of liver disease. In our study, while 30.7% patients with genotype D had normal levels of liver enzymes, the number of patients with the same genotype across advancing stages of liver disease comprised 69.23% (*P* < 0.001). These findings suggest that genotype D correlates with advancing liver disease in this region.

On the other hand in 80% cases genotype F was found to be associated with mild to moderate liver disease. In studies by Livingston et al. [38] and Sánchez-Tapias et al. [39], genotype F was associated with severe infection and HCC development in younger population. Genotype F in our study was found to be strongly associated with HBeAg positivity with odds ratio being 1.9 and risk ratio 1.39. Liver enzymes in genotype F in comparison to genotype D were lower. All the cases of genotype F had NNI as against 60% genotype D cases.

In our study we could not reach any conclusion regarding the severity of genotype A as only one patient was genotyped as A although he had severe liver disease. Kumar et al. [23] reported that genotype A was more severe than D, being significantly more associated with high alanine transaminase levels, HBeAg positivity, anti-HBe negativity, and cirrhosis.

36 (39.56%) genotype D patients in this study had necrotising inflammation. None of the genotype F patients developed necrotising inflammation pointing to the milder form of disease associated with genotype F.

However, 10 (9.34%) samples still did not yield a PCR product. Similarly in a study done by Vivekanandan et al. [18], 5.2% of the strains could not be genotyped. Zekri et al. [37] also mentioned that in multiplex PCR indeterminate samples range from 2.9% to 4.5%. This phenomenon may be the result of intrinsic differences in the sensitivities of these PCRs or the result of the difference in the region of the HBV genome targeted. This may be due to the presence of infection with multiple genotypes or the viral load in these samples was not sufficient enough for the detection limit of these amplification protocols.

DNA sequencing and phylogenetic analysis confirmed genotype D in 10 of our representative samples. A clustering of genotype D in our study was observed with genotype D from India and Syria, D4 of Italy, D2 of Japan, D5 of Poland, and D6 of Germany [17]. The diversity in the clustering in our study may be due to the fact that population groups of Northern, Western, and Eastern India ethnically are of Caucasoid origin, who speak Indo-European languages and show close genetic affinities with populations of Eurasia and Europe [40]. Our study is amongst the few in North India confirming genotypes by DNA sequencing and tracing the roots of our samples [23].

Emergence of genotype F in India merits further study regarding its clinical implications and treatment modalities. Our study suggests it causes milder disease. Information about HBV genotypes can direct a clinician towards more informed management of HBV patients. Long term follow-up studies are required to understand the clinical, therapeutic and epidemiological differences among HBV genotypes especially genotype F in the Indian subcontinent.

## Figures and Tables

**Table 1:** Clinical and biochemical grading of severity of liver disease in different genotypes.

Clinical grading	Genotype D	Genotype F
Mild (%)	13 (14.3)	2 (40)
Moderate (%)	26 (28.6)	2 (40)
Severe (%)	52 (57.1)	1 (20)
Biochemical grading		
Mild (%)	22 (24.1)	1 (20)
Moderate (%)	21 (23.1)	1 (20)
Severe (%)	20 (21.9)	1 (20)

**Table 2:** Demographic profile, hepatitis B and antigen (HBeAg) status, and biochemical profile of patients with different HBV genotypes.

Variable	Genotype D (*n* = 91)	Genotype F (*n* = 5)	Genotype A (*n* = 1)	Nontypable (*n* = 10)
Age, mean years ± SD	34.19 ± 15.01	29.8 ± 12.17	40	38.18 ± 21.87
Ratio of male to female subjects	2.6 : 1	4 : 1	1 : 0	4 : 6
HBeAg positive, no (%)	40 (43.9)	3 (60)	1 (100)	4 (40)
HBeAg negative, no (%)	51 (56.04)	2 (40)	0	6 (60)
Anti-HBc-IgM, no (%)	17 (18.7)	1 (20)	1 (100)	2 (20)
ALT (IU/L) (normal 2–15)	47.61 ± 45.36	23.4 ± 11.54	60	52.90 ± 49.43
AST (IU/L) (normal 2–20)	45.28 ± 44.00	21.8 ± 5.35	90	52.81 ± 41.93
PT-INR	2.40 ± 2.44	1.86 ± 0.79	4.3	2.56 ± 1.79
MELD	19.52 ± 10.57	17 ± 7.41	27	24.18 ± 11.10
Nonnecrotising inflammation (NI)	55 (60.4)	5 (100)	0	6 (60)
Necrotising inflammation (NNI)	36 (39.5)	0	1 (100)	4 (40)
